# *MICA* and *KIR*: Immunogenetic Factors Influencing Left Ventricular Systolic Dysfunction and Digestive Clinical Form of Chronic Chagas Disease

**DOI:** 10.3389/fimmu.2021.714766

**Published:** 2021-08-13

**Authors:** Christiane Maria Ayo, Reinaldo Bulgarelli Bestetti, Eumildo de Campos Junior, Luiz Sérgio Ronchi, Aldenis Albaneze Borim, Cinara Cássia Brandão, Luiz Carlos de Matttos

**Affiliations:** ^1^Immunogenetics Laboratory, Molecular Biology Department, Medicine School in São José do Rio Preto, São José do Rio Preto, Brazil; ^2^Department of Cardiology and Cardiovascular Surgery, Medicine School in São José do Rio Preto, São José do Rio Preto, Brazil; ^3^Surgery Department, Medicine School in São José do Rio Preto, São José do Rio Preto, Brazil

**Keywords:** MHC class I, KIR receptors, Chagas disease, chronic chagasic heart disease, chagasic megacolon, chagasic megaesophagus

## Abstract

Tissue damage observed in the clinical forms of chronic symptomatic Chagas disease seems to have a close relationship with the intensity of the inflammatory process. The objective of this study was to investigate whether the *MICA* (*MHC class I-related chain A*) and *KIR* (*killer cell immunoglobulin-like receptors*) polymorphisms are associated with the cardiac and digestive clinical forms of chronic Chagas disease. Possible influence of these genes polymorphisms on the left ventricular systolic dysfunction (LVSD) in patients with chronic Chagas heart disease was also evaluated. This study enrolled 185 patients with positive serology for *Trypanosoma cruzi* classified according to the clinical form of the disease: cardiac (n=107) and digestive (n=78). Subsequently, patients with the cardiac form of the disease were sub-classified as with LVSD (n=52) and without LVSD (n=55). A control group was formed of 110 healthy individuals. Genotyping was performed by polymerase chain reaction-sequence specific oligonucleotide probes (PCR-SSOP). Statistical analyzes were carried out using the Chi-square test and odds ratio with 95% confidence interval was also calculated to evaluate the risk association. MICA-129 allele with high affinity for the NKG2D receptor was associated to the LVSD in patients with CCHD. The haplotype MICA*008~HLA-C*06 and the KIR2DS2^-^/KIR2DL2^-^/KIR2DL3^+^/C1^+^ combination were associated to the digestive clinical form of the disease. Our data showed that the *MICA* and *KIR* polymorphisms may exert a role in the LVSD of cardiac patients, and in digestive form of Chagas disease.

## Introduction

Chagas disease, resulting from infection by the protozoan *Trypanosoma cruzi*, is a neglected disease that affects about 6-7 million people worldwide, especially in Latin America (1, 2). The acute phase of Chagas disease is usually asymptomatic; when symptomatic, symptoms usually are mild and unspecific (2). Left untreated, the disease progresses relentless to the chronic stage. About 60–70% of these individuals will never present clinical manifestation in the chronic phase, and the only evidence of the disease is positive serology (the so-called indeterminate form of chronic Chagas disease) (1). However, chronically infected individuals may develop clinical manifestations of this disease with irreversible lesions of some organs. After 20 years of infection, about 30% develop chronic Chagas heart disease (CCHD), which is clinically manifested by ventricular arrhythmia (3), thromboembolism (1), sudden cardiac death (1), chronic heart failure (1) and precordial chest pain with normal coronary arteries (4). Ten per cent of Chagas patients present the digestive form of the disease, characterized mainly by dilatations of the esophagus and/or colon (5), cardio-digestive form or, less common, neurological alterations (2).

In CCHD, an important feature is the interplay of autonomic dysfunction, microvascular abnormalities, and autoimmunity (6), which leads to chronic inflammation and reparative fibrosis throughout the myocardium. This can result in the remodeling of the left ventricle, which ultimately leads to left ventricular systolic dysfunction (LVSD). In the digestive form of the disease, the main characteristic is denervation resulting from the destruction of the myenteric plexus (7). The disease causes autonomic nervous system injury throughout all gastrointestinal tract and can occur anatomical and functional abnormalities, mainly related to the lower esophageal sphincter and colon (1). This variation in clinical forms of chronic Chagas disease could be related to the virulence and genetic heterogeneity of parasite, to host genetic factors and differences in immune response (8). The Discrete Typing Units-specific (DTUs) recognition of parasite by cell of the immune system could also result in the differential immune responses (9).

The pathogenic mechanisms involved in the clinical forms of Chagas disease can be explained, at least in part, by the persistence of the parasite maintaining the inflammation (10, 11), and by autoimmunity due to immune response against its own antigens resulting in tissue damage (12). Natural killer (NK) and T cells play a key role in responding to *T. cruzi* infection and the development of clinical forms of Chagas disease (13, 14). These cells are present in focal inflammatory infiltrations observed in both the heart and the digestive tract (14–17). However, the inappropriate expression of NK cell and T cell ligands or receptors induces the autoreactive effector function of these cells and may trigger autoimmune mechanisms (18, 19).

The *MICA* gene, located on chromosome 6p21.33, is responsible for encoding MICA molecules, which are expressed on the cell surface of epithelium, fibroblasts, keratinocytes, endothelial cells and monocytes under stress conditions (20), but its expression is higher in cells of epithelial origin (20, 21). These molecules are recognized by Tαβ, Tγδ and NK cells by the NKG2D receptor (18). The formation of the MICA-NKG2D complex results in a cell-signaling cascade that ends with the target cell lysis process (22). In addition, *KIR* genes are responsible for encoding KIR receptors, which are involved in the regulation of the effector response of NK cells. KIR receptors recognize HLA class I molecules present on the surface of the target cells (23). *KIR* comprise a family of 15 genes located on chromosome 19q13.4, classified as activators (*KIR2DS* and -*3DS*) and inhibitors (*KIR2DL* and -*3DL*) of NK cells and two pseudogenes (*KIR2DP1* and -*3DP1*) (24).

The aim of this study was to investigate whether *MICA* and *KIR* polymorphisms are associated with the cardiac and digestive clinical forms of chronic Chagas disease. Furthermore, the possible association of these gene polymorphisms with LVSD, the powerful independent predictor of all-cause mortality in patients with CCHD (1), was also evaluated because *MICA* alleles and certain *KIR* genes and their HLA ligands may be related to the clinical forms of Chagas disease (25–27). To the best of our knowledge, this appears to be the first study to evaluate the association of *KIR* with digestive clinical form of chronic Chagas disease and with LVSD in patients with CCHD.

## Materials and Methods

As far as possible, the STrengthening the REporting Genetic Association Studies (STREGA) (28) criteria were adopted in the design of this study.

### Ethical Considerations and Subject Selection

This study was approved by the Research Ethics Committee of the São José do Rio Preto Medical School (FAMERP - # 009/2011), and the experiments were carried out in accordance with the approved regulations. An informed consent form was signed by all participants.

This was a cross-sectional, case-control study. Between January 2011 and December 2012, a total of 185 unrelated male and female patients serologically diagnosed with chronic Chagas disease were selected from those who sought treatment at the Cardiomyopathy Outpatient and General Surgery Services of Hospital de Base of the Fundação Faculdade Regional de Medicina (HB-FUNFARME), São José do Rio Preto, SP, Brazil, a tertiary referral center for treating patients with chronic Chagas disease. The control group for the genic and allele frequency analyses consisted of 110 male and female blood donors, eligible for donation, recruited at the Blood Bank in São José do Rio Preto, SP, Brazil. They were tested for Chagas, hepatitis B and C, HIV, syphilis, and HTLV I/II.

All subjects were classified as being from a mixed ethnic population due to high miscegenation of the Brazilian population (29), although they self-reported themselves as European descent, mixed African together with European descent, and African descent. The probability of variations in the allele frequencies due to ancestry background was minimized by matching individuals from similar ancestry backgrounds. Sex and residence in the same geographical areas also were matched during groups selection to avoid bias in the results.

The following inclusion and exclusion criteria were adopted for the patient group: Inclusion - Positive laboratory diagnosis of *T. cruzi* infection, being in the chronic phase of the disease at the time of the study and resident in a municipality in the northwestern region of the state of Sao Paulo. Exclusion - Under 18 years of age, suffering other infectious or parasitic diseases, with any concomitant disease that may induce the onset of chronic heart disease, pregnant women, and patients with any type of mental deficiency. The inclusion criteria for the control group were as follows: eligible for donation, asymptomatic and living in the same geographical region as the patients.

### Clinical Characterization of the Patients

All patients underwent 12-lead electrocardiogram, 2-dimensional echocardiogram and chest X-rays. Patients suspected of having the digestive form of the disease underwent anorectal manometry, X-rays of the opaque enema, esophageal manometry, and X-rays of the esophagus. The diagnosis of chronic Chagas heart disease was based on abnormalities in the resting ECG (right bundle branch block, left anterior fascicular block, left ventricular hypertrophy, left atrial dilatation), which can be found in the presence of a normal 2-D echocardiography. Also, patients were considered to have chronic Chagas heart disease in the presence of abnormalities in the 2-D echocardiogram (right ventricular dilatation, left atrial dilatation, left ventricular dilatation, segmental wall abnormalities, and LVSD). Such alterations are consistent with chronic Chagas heart disease (30). Patients with esophageal disorders at manometry or dilatation at radiological study, as well as those with dilatation of the colon at barium enema were considered to have chronic Chagas digestive disease (7). Patients with a mixed form of the disease (cardiac and digestive) were excluded.

Moreover, CCHD patients were subdivided into two groups according to their left ventricular ejection fraction (LVEF), the powerful independent predictor of all-cause mortality (1), according to the Teichholz method: “Patients without LVSD” for those diagnosed with LVEF ≥60% and “patients with LVSD” for those with LVEF <60% (31).

### Biological Samples

Blood samples were collected into tubes containing EDTA anticoagulant for genomic DNA extraction, that it was made using the PureLinkTM Genomic DNA Mini Kit (Invitrogen, Carlsbad, California, USA) according to the manufacturer’s instructions. The evaluation of concentration and purity of DNA samples was made using the ratio of the absorbance at optical densities (OD) of 260 and 280 nm with Epoch™ equipment (BioTek, Winooski, Vermont, USA). Blood samples were also collected into tubes without anticoagulant to obtain serum for laboratory diagnosis.

### Serology for *T. cruzi*

The laboratory diagnosis of *T. cruzi* infection in patients and controls were made based on the 2nd Brazilian Guidelines for Chagas Diseases (31) and two positive tests were used to establish the diagnosis: enzyme-linked immunosorbent assay (ELISA) using the ELISAcruzi immunoassay (bioMerieux SA Brazil) and indirect immunofluorescence using the Imuno-Com (Wama Diagnóstica, Brazil), following the manufacturer’s instructions on both techniques. Positive and negative controls were included in all reactions and the samples were tested in duplicate.

### MICA, KIR, and HLA Genotyping

*MICA*, *KIR* and *HLA* class I (HLA-A, -B and -C) were genotyped by Polymerase Chain Reaction-Sequence Specific Oligonucleotide Probes protocols with Luminex technology (One Lambda Inc., Canoga Park, CA, USA) according to the manufacturer’s instructions. Hybridization was detected by flow cytometry (LABScanTM 100 flow analyzer) and the data was interpreted using a computer program (HLA Fusion 2.0 Research, One Lambda).

HLA-KIR ligand specificities belonging to the groups C1, C2, Bw4 (Bw4-80Ile and Bw4-80Thr) and HLA-A*03/-A*11 were considered according to Carr et al. (32), Thananchai et al. (33), and Kulkarni et al. (34). *KIR* haplotypes AA and BX (BB and AB) were defined based on the number of the genes encoding activating receptors (according to http://www.allelefrequencies.net). Binding affinities to NKG2D, attributed to the amino acid position 129 in the *MICA* gene, were determined by Steinle et al. (35) and Karacki et al. (36), based on amino acid composition (methionine, high; valine, low – in respect to the rs1051792 polymorphism). Due to physical proximity with *HLA-B*, *MICA* ambiguities were solved by linkage disequilibrium (LD) between them.

### Statistical Analysis

*KIR* and MICA-129 frequencies were obtained by direct counting. The ARLEQUIN software version 3.11 (http://cmpg.unibe.ch/software/arlequin3) was used to calculate the allele groups (HLA and MICA) and haplotype frequencies. The Hardy-Weinberg equilibrium was verified according to the method described by Guo & Thompson (37) for KIR2DL2/3 and KIR3DL1/S1, and for MICA and HLA alleles. The relative LD (Δ’) was calculated according to the Imanishi method (38). Comparisons between groups of patients were attained using the chi-square test with Yates’ correction or Fisher’s exact test using the statistics program OpenEpi version 3.01 (http://www.openepi.com/Menu/OE_Menu.htm). The genetic associations were measured by OR (Odds Ratio) and the 95% confidence interval (95% CI). The unpaired t-test was used to compare the mean ages. P-values < 0.05, corrected by the Bonferroni inequality method for multiple comparisons (Pc), were considered statistically significant.

## Results

The clinical characteristics of patients and controls are shown in **Table 1**. Of the 185 patients (88 men and 97 women; mean age of 65.2 ± 10.4 years) with positive serology for *T. cruzi*, 78 (42.2%) (35 men and 43 women; mean age of 65.8 ± 10.8 years) were considered to have digestive form of Chagas disease and 107 (57.8%) (53 men and 54 women; mean age of 64.7 ± 10.2 years) were considered to have cardiac form of Chagas disease. Of the 78 patients with digestive form, 21 (26,9%) (5 men and 16 women; mean age of 64.3 ± 10.3 years) had megacolon (MGC), 44 (56.4%) (23 men and 21 women; mean age of 66.8 ± 11.2 years) had magaesophagus (MGE), and 13 (16.6%) (7 men and 6 women; mean age of 64.7 ± 10.8 years) had MGC and MGE. Of the 107 patients with CCHD, 52 (48.6%) (30 men and 22 women; mean age of 64.9 ± 10.3 years) had LVSD, whereas the remaining 55 (51.4%) (23 men and 32 women; mean age of 64.5 ± 10.1 years) did not. The control group was comprised by 110 healthy individuals (47 man and 63 women; mean age of 54.4 ± 5.1 years). The difference between the mean age of patient groups with chronic Chagas disease (cardiac or digestive) and the control group was statistically significant (p <0.00001).

**Table 1:** Characteristics of the chronic Chagas disease patients and control individuals.

Characteristic	Patients	Control
	(n = 185)	(n = 110)
Age* (Mean ± SD)	65.2 ± 10.4	54.4 ± 5.1
Sex (n as %)		
*Male*	88 (47.6)	47 (42.7)
*Female*	97 (52.4)	63 (57.3)
Ancestry backgrounds** (n as %)		
*European descent*	92 (49.7)	61 (55.4)
*Mixed African with together European descent*	78 (42.2)	42 (38.2)
*African descent*	15 (8.1)	7 (6.4)
Clinical forms (n as %)		–
Cardiac	107 (57.8)	
*With LVSD*	52 (48.6)	
*Mean ± SD*	64.9 ± 10.3	
*sex (n) male/female*	30/22	
*Without LVSD*	55 (51.4)	
*Mean ± SD*	64.5 ± 10.1	
*sex (n) male/female*	23/32	
Digestive	78 (42.2)	
*With MGC*	21 (26.9)	
*Mean ± SD*	64.3 ± 10.3	
*sex (n) male/female*	5/16	
*With MGE*	44 (56.4)	
*Mean ± SD*	66.8 ± 11.2	
*sex (n) male/female*	23/21	
*With MGC+MGE*	13 (16.6)	
*Mean ± SD*	64.7 ± 10.8	
*sex (n) male/female*	7/6	

*P-value <0.00001 when mean age of patients group and subgroups of patients (cardiac and digestive) were compared to the control group. t = 10.1 (Patients vs. Control). t = 9.4 (Cardiac vs. Control). t = 9.6 (Digestive vs. Control). t = 8.7 (With LVSD vs. Control). t = 8.5 (Without LVSD vs. Control). t = 6.7 (With MGC vs. Control). t = 9.4 (With MGE vs. Control). t = 5.8 (With MGC + MGE vs. Control). **All subjects were classified as being from a mixed ethnic population due to high miscegenation of the Brazilian population (29). LVSD, left ventricular systolic dysfunction; MGC, megacolon; MGE, magaesophagus; SD, Standard deviation.

*MICA, KIR* (*KIR2DL2/3* and *KIR3DL1/S1*) and *HLA* class I frequencies of the control group were in Hardy-Weinberg equilibrium (p-value > 0.05).

No significant differences were found in the distribution of *MICA* alleles between the groups. Moreover, statistically significant differences were not found for both the MICA-129 alleles with high affinity and MICA-129 alleles with low affinity for the NKG2D receptor between patients with the cardiac and digestive forms of Chagas disease. However, the MICA-129 alleles with high affinity showed a risk association to LVSD in patients with CCHD. The frequency was higher in patients with LVSD than in patients without LVSD (OR = 1.97; CI = 1.09-3.33; p-value = 0.02; Pc = 0.04) and controls (OR = 1.98; CI = 1.19-3.28; p-value = 0.007; Pc = 0.01). No significant differences were found for the MICA-129 alleles between patients without LVSD and controls (**Table 2**).

**Table 2:** Distribution of MICA alleles in patients with the digestive or cardiac forms of chronic Chagas disease, in patients with LVSD and without LVSD and in control individuals.

MICA		Digestive (n = 78)	Cardiac (n = 107)	With LVSD (n = 52)	Without LVSD (n = 55)	Control (n = 110)
		n (%)	n (%)	n (%)	n (%)	n (%)
Alleles	Position 129					
*001	met	2 (1.3)	6 (2.8)	2 (1.9)	4 (3.6)	7 (3.2)
*002	met	33 (21.2)	40 (18.7)	23 (22.1)	17 (15.5)	40 (18.2)
*004	val	26 (16.7)	25 (11.7)	11 (10.5)	14 (12.7)	33 (15.0)
*006	val	2 (1.3)	0 (0.0)	0 (0.0)	0 (0.0)	1 (0.5)
*007	met	4 (2.6)	9 (4.2)	6 (5.8)	3 (2.7)	6 (2.7)
*008	val	41 (26.3)	51 (23.8)	22 (21.2)	29 (26.4)	61 (27.7)
*009	val	15 (9.6)	26 (12.1)	12 (11.5)	14 (12.7)	31 (14.1)
*010	val	8 (5.1)	8 (3.7)	3 (2.9)	5 (4.5)	12 (5.4)
*011	met	4 (2.6)	6 (2.8)	5 (4.8)	1 (0.9)	6 (2.7)
*012	met	1 (0.6)	0 (0.0)	0 (0.0)	0 (0.0)	0 (0.0)
*015	met	1 (0.6)	3 (1.4)	2 (1.9)	1 (0.9)	2 (1.0)
*016	val	3 (1.9)	3 (1.4)	0 (0.0)	3 (2.7)	4 (1.8)
*017	met	5 (3.2)	6 (2.8)	2 (1.9)	4 (3.6)	2 (1.0)
*018	met	6 (3.8)	12 (5.6)	7 (6.7)	5 (4.5)	7 (3.1)
*019	val	1 (0.6)	7 (3.3)	4 (3.8)	3 (2.7)	4 (1.8)
*024	val	0 (0.0)	1 (0.5)	(0.0)	1 (0.9)	0 (0.0)
*027	val	3 (1,9)	8 (3.7)	2 (1.9)	6 (5.4)	4 (1.8)
*029	met	0 (0.0)	2 (0.9)	2 (1.9)	0 (0.0)	0 (0.0)
*030	met	0 (0.0)	1 (0.5)	1 (1.0)	0 (0.0)	0 (0.0)
*045	met	1 (0.6)	0 (0.0)	0 (0.0)	0 (0.0)	0 (0.0)
MICA-129						
High affinity^§^		57 (36.5)	85 (39.7)	50 (48.1)^a,b^	35 (31.8)^a^	70 (31.8)^b^
Low affinity^§^		99 (63.5)	129 (60.2)	54 (51.9)	75 (68.2)	150 (68.2)

a OR = 1.98; CI = 1.13-3.46; p-value = 0.02; Pc = 0.04 (With LVSD vs. Without LVSD).

b OR = 1.98; CI = 1.23-3.20; p-value = 0.007; Pc = 0.01 (With LVSD vs. Control).

LVSD, left ventricular systolic dysfunction; Met, methionine; Val, valine. ^§^Based on amino-acid composition (methionine, high; valine, low - related to the rs1051792) in position 129 of the MICA gene (35,36). Pc, p-value corrected by the Bonferroni inequality method.

The asterisk symbol [*] is used in the official nomenclature of MHC genes to designate that the method of definition was molecular.

**Table 3** shows the frequency of the MICA~HLA-B and MICA~HLA-C haplotypes that presented a p-value < 0.05 in the comparison of the groups. The haplotypes MICA*002~HLA-C*07 (OR = 4.30; CI = 1.14-16.17; p-value = 0.04; Pc = 1.32) and MICA*008~HLA-C*06 (OR = 13.04; CI = 1.63-104.0; p-value = 0.005; Pc = 0.04) showed a higher frequency in patients with the digestive than in patients with cardiac form of the disease. MICA*008~HLA-C*06 was also more frequent in patients with the digestive than in control group (OR = 4.42; CI = 1.18-16.63; p-value = 0.03; Pc = 0.30). Moreover, the haplotype MICA*008~HLA-B*44 (OR = 3.84; CI = 1.09-13.52; p-value = 0.04; Pc = 0.64) was more frequent in patients with cardiac form than in patients with the digestive form of the disease. However, the significance of these associations was not statistically significant after correcting for multiple comparisons, except for the haplotype MICA*008~HLA-C*06, associated as a factor of risk for digestive form of Chagas disease. There was no significant difference on comparing the MICA~HLA-B and MICA~HLA-C haplotypes between patients with LVSD and those without LVSD. The frequencies of HLA-A, -B and –C alleles were similar between groups.

**Table 3:** Haplotype frequencies of MICA, HLA-B and HLA-C in patients with the digestive or cardiac forms of chronic Chagas disease, in patients with LVSD and without LVSD and in control individuals.

Haplotypes	Digestive (n = 78)	Cardiac (n = 107)	With LVSD (n = 52)	Without LVSD (n = 55)	Control (n = 110)	Δ’ (p-value)
	n (%)	n (%)	n (%)	n (%)	n (%)	
MICA~HLA-B						
*008~*44	3 (1.9)^a^	15 (7.0)^a^	7 (6.7)	8 (7.3)	14 (6,4)	0.25 (0.12)^#^
MICA~HLA-C						
*002~*07	9 (5.8)^b^	3 (1.4)^b^	1 (0.9)	2 (1.8)	4 (1.8)	0.03 (0.79)^##^
*008~*06	9 (5.8)^c^ ^,^ ^d^	1 (0.5)^c^	0 (0.0)	1 (0.9)	3 (1.3)^d^	0.74 (0.06)^##^

a OR = 3.84; CI = 1.09-13.52; p-value = 0.04; Pc = 1.16 (Digestive form vs. Cardiac form).

b OR = 4.30; CI = 1.14-16.17; p-value = 0.04; Pc = 1.24 (Digestive form vs. Cardiac form).

c OR = 13.04; CI = 1.63-104.0; p-value = 0.005; Pc = 0.04 (Digestive form vs. Cardiac form).

d OR = 4.42; CI = 1.18-16.63; p-value = 0.03; Pc = 0.30 (Digestive form vs. Control).

^#^Δ’ (p-value) in patients group with cardiac form of disease. ^##^Δ’ (p-value) in patients group with digestive form of disease. LVSD, left ventricular systolic dysfunction; Pc, p-value corrected by the Bonferroni inequality method; Δ’, relative linkage disequilibrium.

The asterisk symbol [*] is used in the official nomenclature of MHC genes to designate that the method of definition was molecular.

**Table 4** shows the distribution of *KIR* gene frequencies. Cardiac patients presented a higher frequency of *KIR2DS2* activating gene when compared to the patients with the digestive form of the disease (OR = 1.89; CI = 1.05-3.43; p-value = 0.04; Pc = 0.64), but the significance was lost after applying the Bonferroni correction.

**Table 4:** Distribution of *KIR* genes and KIR haplotypes in patients with the digestive or cardiac forms of chronic Chagas disease and, in patients with LVSD and without LVSD and in control individuals.

*KIR* Genes	Digestive (n = 78)	Cardiac (n = 107)	With LVSD (n = 52)	Without LVSD (n = 55)	Control (n = 110)
	n (%)	n (%)	n (%)	n (%)	n (%)
Inhibitory					
*KIR2DL1*	74 (94.9)	103 (96.3)	51 (98.0)	52 (94.5)	108 (98.2)
*KIR2DL2*	36 (46.2)	64 (59.8)	31 (59.6)	33 (60.0)	61 (55.4)
*KIR2DL3*	62 (79.5)	96 (89.7)	46 (88.5)	50 (90.9)	97 (88.1)
*KIR2DL5*	43 (55.1)	64 (59.8)	31 (59.6)	33 (60.0)	68 (61.8)
*KIR3DL1*	75 (96.2)	103 (96.3)	49 (94.2)	54 (98.2)	105 (95.5)
Activating					
*KIR2DS1*	33 (42.3)	42 (39.3)	18 (34.6)	24 (43.6)	46 (41.8)
*KIR2DS2*	30 (38.5)^a^	58 (54.2)^a^	26 (50.0)	32 (58.2)	58 (52.7)
*KIR2DS3*	17 (21.8)	33 (30.8)	17 (32.7)	16 (29.1)	38 (34.5)
*KIR2DS4*	75 (96.2)	101 (94.4)	48 (92.3)	53 (96.4)	104 (94.5)
*KIR2DS5*	26 (33.3)	50 (46.7)	23 (44.2)	27 (49.1)	46 (41.8)
*KIR3DS1*	33 (42.3)	40 (37.4)	18 (34.6)	20 (36.7)	42 (38.2)
Framework and Pseudogenes					
*KIR2DL4*	78 (100.0)	107 (100.0)	52 (100.00)	55 (100.0)	110 (100)
*KIR3DL2*	78 (100.0)	107 (100.0)	52 (100.00)	55 (100.0)	110 (100)
*KIR3DL3*	78 (100.0)	107 (100.0)	52 (100.00)	55 (100.0)	110 (100)
*KIR3DP1*	78 (100.0)	107 (100.0)	52 (100.00)	55 (100.0)	110 (100)
*KIR2DP1*	74 (94.9)	104 (97.2)	51 (98.1)	53 (96.4)	108 (98.2)
Haplotypes					
AA	24 (30.8)	21 (19.6)	12 (23.1)	9 (16.4)	26 (23.6)
BX	54 (69.2)	86 (80.4)	40 (76.9)	46 (83.6)	84 (76.4)

a OR = 1.89; CI = 1.05-3.43; p-value = 0.04; Pc = 0.64 (Cardiac form vs. Digestive form).

LVSD, left ventricular systolic dysfunction; Pc, p-value corrected by the Bonferroni inequality method.

No significant differences were found in the distribution of KIR haplotypes (AA and BX) and in the distribution of KIR-HLA receptor ligand pairs between all groups investigated in this study (**Tables 4** and **5**, respectively). The frequencies of HLA class I ligands of KIR (A3 or A11, Bw4 and Bw4-80Ile, C1 and C2, in homozygosity and heterozygosity) were similar between groups.

**Table 5:** Distribution of KIR and their respective HLA ligands in patients with the digestive or cardiac forms of chronic Chagas disease, in patients with LVSD and without LVSD and in control individuals.

KIR - HLA ligands	Digestive (n = 78)	Cardiac (n = 107)	With LVSD (n = 52)	Without LVSD (n = 55)	Control (n = 110)
	n (%)	n (%)	n (%)	n (%)	n (%)
Inhibitory					
2DL1-C2	63 (80.8)	90 (84.1)	44 (84.6)	46 (83.6)	86 (78.2)
2DL2-C1	33 (42.3)	50 (46.7)	25 (48.1)	25 (45.5)	38 (34.5)
2DL3-C1	53 (67.9)	68 (63.6)	34 (65.4)	34 (61.8)	59 (53.6)
3DL2-A3/A11	19 (24.4)	26 (24.3)	13 (25.0)	13 (23.6)	36 (32.7)
3DL1-Bw4	41 (52.6)	78 (72.9)	36 (69.2)	42 (76.4)	75 (68.5)
2DL1-C2C2	10 (12.8)	25 (23.4)	10 (19.2)	15 (27.3)	22 (20.0)
2DL2-C1C1	3 (3.8)	12 (11.2)	6 (11.5)	6 (10.9)	12 (10.9)
2DL3-C1C1	10 (12.8)	13 (12.1)	6 (11.5)	7 (12.7)	20 (18.8)
2DL2/2DL2-C1C2	14 (17.7)	10 (9.3)	5 (9.6)	5 (9.1)	6 (5.5)
2DL2/2DL3-C1C2	16 (20.5)	28 (26.2)	14 (26.9)	14 (25.5)	29 (26.4)
2DL3/2DL3-C1C2	27 (34.6)	27 (25.2)	14 (26.9)	13 (23.6)	23 (20.9)
2DL2/2DL2-C1C1	1 (1.3)	1 (0.9)	1 (1.9)	0 (0.0)	2 (1.8)
2DL2/2DL3-C1C1	2 (2.6)	11 (10.3)	5 (9.6)	6 (10.9)	10 (9.1)
2DL3/2DL3-C1C1	8 (10.3)	4 (3.7)	3 (5.8)	1 (1.8)	8 (7.2)
Activating					
2DS1-C2	27 (34.6)	34 (31.8)	13 (25.0)	21 (38.2)	36 (32.7)
2DS2-C1	26 (33.3)	45 (42.1)	22 (42.3)	23 (41.8)	45 (40.9)
2DS1-C2C2	5 (6.4)	10 (9.3)	4 (7.7)	6 (10.9)	8 (7.2)
2DS2-C1C1	3 (3.8)	8 (7.5)	4 (7.7)	4 (7.3)	7 (6.3)
3DS1-Bw4-80Ile	16 (20.5)	19 (17.8)	9 (17.3)	10 (18.2)	20 (18.8)

Bw4 = HLA-A*23, *24, *32; HLA-B *13, *27, *44, *51, *52, *53, *57, *58. Bw4-80Ile = HLA-A*23, *24, *32; HLA-B*51, *52, *53, *57, *58. Group C1 = HLA-C*01, *03, *07, *08, *12, *14, *16. Group C2 = HLA-C*02, *04, *05, *06, *07, *15, *17, *18. HLA-KIR ligands specificities were considered according to Carr et al. (32), Thananchai et al. (33) and Kulkarni et al. (34). LVSD, left ventricular systolic dysfunction.

The combination between the distribution of activating and inhibitory KIR and their respective HLA ligands is shown in **Table 6**. Both 2DS2^-^/2DL2^-^/2DL3^+^/C1^+^ and 2DS2^-^/2DL3^+^/C1^+^ combinations were more frequent in patients with digestive form than in patients with the cardiac form of the disease (OR = 2.40; CI = 1.28-4.51; p-value = 0.009; Pc = 0.03 and OR = 2.09; CI = 1.15-3.80; p-value = 0.02; Pc = 0.06 respectively) and in control group (OR = 2.26; CI = 1.21-4.20; p-value = 0.01; Pc = 0.04; and OR = 2.10; CI = 1.16-3.80; p-value = 0.02; Pc = 0.06 respectively), but after Bonferroni correction only 2DS2^-^/2DL2^-^/2DL3^+^/C1^+^ combination was associated with the risk of the digestive form of chronic Chagas disease. A significant difference was found for 2DS1^+^/2DL1^+^/C2^+^ combination between patients with LVSD and those without LVSD, but only before p-value correction (OR = 0.35; CI = 0.14-0.85; p-value = 0.03; Pc = 0.09).

**Table 6:** Distribution of activating KIR plus inhibitory KIR and their respective ligands in patients with the digestive or cardiac forms of chronic Chagas disease, in patients with LVSD and without LVSD and in control individuals.

Activating and/or inhibitory KIR and HLA ligands combinations	Digestive (n = 78)	Cardiac (n = 107)	With LVSD (n = 52)	Without LVSD (n = 55)	Control (n = 110)
	n (%)	n (%)	n (%)	n (%)	n (%)
KIR-C1					
2DS2+/2DL2-/C1+	2 (2.5)^a^	11 (10.3)^a^	4 (7.7)	7 (12.7)	6 (5.4)
2DS2-/2DL2+/C1+	7 (9.0)	14 (13.1)	7 (13.5)	7 (12.7)	5 (4.5)
2DS2+/2DL3-/C1+	15 (19.2)	10 (9.3)	6 (11.5)	4 (7.3)	9 (8.2)
2DS2-/2DL3+/C1+	41 (52.6)^b,c^	37 (34.6)^b^	18 (34.6)	19 (34.5)	38 (34.5)^c^
2DS2+/2DL2-/2DL3-/C1+	0 (0.0)	0 (0.0)	0 (0.0)	0 (0.0)	0 (0.0)
2DS2-/2DL2-/2DL3+/C1+	34 (43.6)^d,e^	26 (24.3)^d^	14 (26.9)	12 (21.8)	28 (25.5)^e^
2DS2+/2DL2-/2DL3+/C1+	1 (1.3)	9 (8.4)	4 (7.7)	5 (9.1)	4 (3.6)
2DS2-/2DL2+/2DL3+/C1+	7 (9.0)	13 (12.1)	7 (13.5)	6 (10.9)	5 (5.4)
2DS2+/2DL2+/2DL3-/C1+	14 (17.9)	10 (9.3)	6 (11.5)	4 (7.3)	8 (7.3)
2DS2+/2DL2+/2DL3+/C1+	11 (14.1)	27 (25.2)	13 (25.0)	14 (25.5)	29 (26.3)
KIR-C2					
2DS1+/2DL1-/C2+	0 (0.0)	1 (0.9)	0 (0.0)	1 (1.8)	2 (1.8)
2DS1-/2DL1+/C2+	36 (46.2)	55 (51.4)	30 (57.7)	25 (45.5)	50 (45.6)
2DS1+/2DL1+/C2+	26 (33.3)	32 (29.9)	10 (19.2)^f^	22 (40.0)^f^	32 (29.1)
KIR-BW4-80Ile					
3DS1+/3DL1+/BW4-80Ile+	15 (19.2)	18 (16.8)	8 (15.4)	10 (18.2)	16 (14.4)
3DS1+/3DL1-/BW4-80Ile+	1 (1.3)	1 (0.9)	1 (1.9)	0 (0.0)	1 (0.9)
3DS1-/3DL1+/BW4-80Ile+	28 (35.9)	38 (35.5)	17 (32.7)	21 (38.2)	39 (35.5)

a OR = 4.35; CI = 0.94-20.24; p-value = 0.07 (Cardiac form vs. Digestive form).

b OR = 2.09; CI = 1.15-3.80; p-value = 0.02; Pc = 0.06 (Digestive form vs. Cardiac form).

c OR = 2.10; CI = 1.16-3.80; p-value = 0.02; Pc = 0.06 (Digestive form vs. Control).

d OR = 2.40; CI = 1.28-4.51; p-value = 0.009; Pc = 0.03 (Digestive form vs. Cardiac form).

e OR = 2.26; CI = 1.21-4.20; p-value = 0.01; Pc = 0.04 (Digestive form vs. Control).

f OR = 0.35; CI = 0.14-0.85; p-value = 0.03; Pc = 0.09 (With LVSD vs. Without LVSD).

Bw4 = HLA-A*23, *24, *32; HLA-B *13, *27, *44, *51, *52, *53, *57, *58. Bw4-80Ile = HLA-A*23, *24, *32; HLA-B*51, *52, *53, *57, *58. Group C1 = HLA-C*01, *03, *07, *08, *12, *14, *16. Group C2 = HLA-C*02, *04, *05, *06, *07, *15, *17, *18. HLA-KIR ligands specificities were considered according to Carr et al. (32), Thananchai et al. (33) and Kulkarni et al. (34). LVSD, left ventricular systolic dysfunction; Pc, p-value corrected by the Bonferroni inequality method.

## Discussion 

The data obtained in this study provide evidence that broadens and reinforces our knowledge about the influence of the *MICA* and *KIR* genetic variants and their interactions with HLA class I molecules in the different clinical forms of chronic Chagas disease. In particular, MICA-129 allele with high affinity for the NKG2D receptor was associated to the LVSD. The haplotype MICA*008~HLA-C*06 and the KIR2DS2^-^/KIR2DL2^-^/KIR2DL3^+^/C1^+^ combination were associated to the digestive clinical form of the disease.

MICA and KIR molecules are directly involved in the activation and regulation of NK cell activity. The effector mechanisms of NK cells act on the target cell when there is no recognition of HLA class I molecules by KIR receptors and/or when MICA molecules are recognized by the NKG2D receptor (17), hypotheses known as “missing self” and “induced self” respectively (39). Furthermore, MICA molecules act as co-stimulators of Tαβ (CD8^+^ and subsets of CD4^+^) and Tγδ cells (40). It is known that NK and T cells are part of the inflammatory infiltrate found in the cardiac and digestive forms of chronic Chagas disease (10, 14–17). Due to the polymorphic nature of the *MICA* and *KIR* genes, the proteins encoded by them are highly diverse (18, 24). As a result, different patterns of proteins may explain the variability in the response of NK and CD8^+^ T cells in different clinical forms of the disease. The interaction of these cells with the target cell exerts a significant role in the initiation and regulation of the innate and adaptive immune responses, and the molecular basis of these cellular interactions seem to be crucial for an efficient and modulated response against the parasite to avoid tissue damage.

The MICA-129 polymorphism was previously reported by our group as risk factors for severe LVSD in patients with CCHD. The met allele (high affinity for the NKG2D receptor) contributed to LVSD under an additive inheritance model; individuals who were homozygous for the met allele had the highest risk of presenting severe LVSD (26). Even though the strategies adopted for the composition of the present study were different from those adopted previously (26), MICA-129 met seems to be a risk factor for LVSD in a population of patients with CCHD from the northwestern region of the state of São Paulo (southeastern Brazil), even that the severity of LVSD has not been considered.

The amino acid methionine at position 129 of the α2 domain of the MICA protein (MICA-129met) has higher affinity for the NKG2D receptor than proteins with the amino acid valine (MICA-129val) in the same position due to a conformational change of the molecule, which may compromise NK cell activation and the co-stimulation of CD8^+^ T lymphocytes (33). Chronic myocarditis has been correlated with the clinical severity of CCHD (41). A high production of cytokines induces migration of large numbers of inflammatory cells into the myocardium, which act as an effector of heart damage (15, 41). The strong affinity formed by the NKG2D-MICA-129met complex may be related to the tissue damage that causes LVSD in patients with CCHD, since the NKG2D-MICA interaction may participate in inflammatory processes with increased cytokine production by NK and TCD8^+^ (18). The involvement of these cells in the immunopathological mechanisms of the cardiac form of Chagas disease can be attributed to their cytotoxic effects, mainly because of the production of cytokines with a Th1 response profile such as interferon-gamma (IFN-γ) (15, 42).

Furthermore, the interaction between NKG2D and the MICA molecules may also favor autoimmune conditions by promoting costimulatory signaling of specific CD8^+^ T cell self-antigens (18). Thus, a milieu of potent immune stimuli may overcome the threshold of activation needed to breach self-tolerance. Autoimmunity is accepted as one of the pathogenic mechanisms responsible for the different clinical manifestations of Chagas disease, as the infectious agent can mimic self-antigens, induce autoreactive cell proliferation or increase the expression of major histocompatibility complex (MHC) and costimulatory molecules in infected cells (43). Associations of CD8^+^ T cells with degenerated ganglion cells has been reported in patients with megacolon (44). These cells were also seen to be related to the destruction of myofibers in heart tissue (12).

This study also showed that the MICA*008~HLA-C*06 haplotype was a risk factor for digestive form of Chagas disease. The MICA*008 allele carries the microsatellite A5.1 characterized by the insertion of a guanine nucleotide after the second GCT (GGCT) repeat, which generates a stop codon in the exon that encodes the transmembrane domain and results in a truncated form of the protein (45). Furthermore, the MICA*008 allele has the amino acid valine at position 129 of the protein. Thus, the risk factor for the digestive form of the disease may be related to the expression of the mutated protein, A5.1, which would affect its recognition by NKG2D receptors. The result of a weak MICA-129val interaction could lead to an increased expression of NKG2D as in autoimmune diseases (46, 47), and favor interactions with other ligand binding proteins such as UL16-binding proteins (ULBPs) (48). This creates an environment rich in cytokines that enhances the cytotoxic activity of CD8^+^ T cells and NK cells and therefore the production of IFN-γ, again favoring the autoimmunity condition (46, 47). It is also known that MICA stimulates Tγδ cells in the intestinal mucosa, a phenomenon that could be related to the digestive form of the disease (49).

Class I HLA molecules play a crucial role in determining the individual immune response through the peptide presentation of pathogens endogenous to CD8^+^ T cells. Thus, peptide affinity for MHC class I appears to be determinant for an effective antigen-directed response, as this interaction may result in distinct patterns of CD8^+^ T cell responses (50). As the associations involving *MICA* and *HLA* alleles were not separately observed, it is difficult to determine the primary associated locus within the haplotype. It is known that some *MICA* alleles may be intimately linked to other alleles also responsible for the association, such as HLA, due to the relatively close physical proximity between these loci and thus, when combined they exert a synergistic effect (51). Thus, both alleles may be exerting an influence on the Chagas disease megacolon and/or magaesophagus.

The data from this study indicate possible risk association related to the inhibitory KIR2DL3 and its C1 ligand in the absence of both KIR2DL2 and KIR2DS2 (2DS2^-^/2DL2^-^/2DL3^+^/C1^+^) in the digestive form of chronic Chagas disease, which has never been previously published. The interaction between KIR and HLA class I molecules, KIR ligands, is required for the functional activity of NK cells, so that the presence of one, but not the other, is not sufficient to influence their function (34). It is known that different KIR receptors (inhibitors and activators) have affinity for the same ligand, however the affinity for the inhibitory receptor seems to be stronger than for its homolog activator (52). There is also a well-established inhibition affinity scale for the KIR2DL-HLA-C pairs: KIR2DL1-C2 has the greatest inhibitory potential, followed by KIR2DL2-C1 and KIR2DL3-C1 (53).

KIR2DL2 and KIR2DL3 segregate as alleles of the same locus and both recognize C1 group ligands. However, these receptors present qualitative differences in their functional effect and clinical influence (54). The main feature of the digestive form of the disease is denervation resulting from the reduction in the number of neurons (10). The neurons death is associated with immune system mononuclear cell adherence and lyses (5). NK cells and cytotoxic lymphocytes are prevalent in the esophagus and colon of chronic chagasic patients (8). In patients with megacolon, NK cells are located between muscle layers or in proximity to the myenteric plexus (44). Thus, the weak inhibitory signal generated by the absence of KIR2DL2, that is, the homozygosity of KIR2DL3 in the presence of the C1 ligand found in the 2DS2^-^/2DL2^-^/2DL3^+^/C1^+^ combination, appears not to be sufficient to inhibit the effector function of NK cells and protect against the exacerbated inflammation that results in tissue lesions of the gastrointestinal tract. KIR2DL-HLA-C inhibitor pairs have been shown to exert influence on inflammatory bowel diseases (55, 56).

In addition to the results of this study, the KIR2DS2-C1 activating pair, in the absence of its inhibitory homolog, KIR2DL2, independently of the presence or absence of KIR2DL3, has been shown to constitute a possible risk factor for Chagas disease and CCHD in patients from a population in southern Brazil (27). In this study, the 2DS2^+^/2DL2^-^/C1^+^ combination showed only a tendency of a positive association with the cardiac form. However, the possible distinction of results can be explained by the influence of ethnicity on the distribution of *KIR* genes and HLA alleles in patients chronically infected by *T. cruzi* from different Brazilian regions. Other methodological variables may have contributed to the observed differences.

The inflammatory infiltrate could be responsible for tissue damage in both forms of chronic Chagas disease (5). NK cells can increase T cell response activity through the production of IFN-γ and both can migrate to the region of inflamed tissues. In addition, NK cell-mediated target cell death also affects T cell responses, possibly by decreasing parasite burden and/or because target cell debris can promote the cross-presentation of antigens to CD8^+^ T cells (57). According to Sathler-Avelar et al. (13), strong and uncontrolled activation of NK cells as well as proinflammatory monocytes may result in the tissue damage observed in the clinical manifestations of chronic Chagas disease. Baseline levels of NK, NKT, and CD4^+^ CD25^high^ cells, increased expression of activated CD8^+^ T cells associated with failed immunoregulation mechanisms were associated with cardiac symptoms of chronic Chagas disease (58). Thus, NK and T cells need to be properly activated to become competent in performing their functional activities without causing tissue damage, and the molecular interactions between receptors and stimulatory and costimulatory molecules and the production of cytokines in the activation process of these cells may be closely related to the immunophysiological processes of CCHD and Chagas disease megacolon and magaesophagus. Of course, there are other cells and other immunophysiological mechanisms involved in the pathogenesis of both cardiac and digestive Chagas disease.

There was a difference in respect to age, with the mean age of the control group being lower than the mean age of the patient groups. Sixty-seven years is the age maximum limit for blood donation in Brazil (59), which results in a younger population compared to the patients with Chagas disease. Additionally, the high mean age of patients is because the chronic clinical forms of Chagas disease develop many years after initial infection, usually after two decades (1, 2, 31). Besides age, can presume that they were not exposed to the infection by *T. cruzi*, this fact also could be a bias. However, blood donors are useful as control groups to compare the frequencies of the alleles and genes with patient groups since they are representative of the general population (60).

In conclusion, our study shows that *MICA* and *KIR* polymorphisms are associated with LVSD in patients with CCHD as well as in digestive Chagas disease, respectively. These findings may be related to the inflammatory activity carried out by NK and T cells. However, further studies are needed, such are histopathological analysis and cytotoxicity assays, to further clarify if such associations play a causal role in LVSD and in MGC and/or MGE. Studies involving other populations, especially those comprised of patients with LVSD without infection by *T. cruzi*, should also be performed.

## Data Availability Statement

The original contributions presented in the study are included in the article. Further inquiries can be directed to the corresponding author.

## Ethics Statement

This study was approved by the Research Ethics Committee of the Medicine School in São Jose do Rio Preto (FAMERP - # 009/2011) and the experiments were carried out in accordance with the approved regulations. An informed consent form was signed by all participants. The patients/participants provided their written informed consent to participate in this study.

## Author Contributions 

CA performed the genotyping of HLA class I, KIR and MICA, analyzed the results and performed the statistical analyzes. CA and RB wrote the paper. CB contributed to the laboratory diagnosis of Chagas disease. CB and LM guided and followed the development of the research and sought financial support. CA, RB, CB and LM reviewed the article. RB, ECJ, LR doctors responsible for clinical diagnosis of chronic Chagas disease. All authors contributed to the article and approved the submitted version.

## Funding

This study was supported by Fundação de Amparo à Pesquisa do Estado de São Paulo (FAPESP) [grant numbers: 2013/06580-9 and 2011/08075-4] and Coordenação de Aperfeiçoamento de Pessoal de Nível Superior –Brasil (CAPES) –Finance Code 001. 

## Author Disclaimer

The opinions, assumptions, and conclusions or recommendations expressed in this material are the responsibility of the authors and do not necessarily reflect the views of FAPESP. The funders had no role in study design, data collection and analysis, decision to publish, or preparation of the manuscript.

## Conflict of Interest

The authors declare that the research was conducted in the absence of any commercial or financial relationships that could be construed as a potential conflict of interest.

## Publisher’s Note

All claims expressed in this article are solely those of the authors and do not necessarily represent those of their affiliated organizations, or those of the publisher, the editors and the reviewers. Any product that may be evaluated in this article, or claim that may be made by its manufacturer, is not guaranteed or endorsed by the publisher.

## References

[1] Bocchi EA Bestetti RB Scanavacca MI Cunha Neto E Issa VS. Chronic Chagas Heart Disease Management: From Etiology to Cardiomyopathy Treatment. J Am Coll Cardiol (2017) 70:1510–24. 10.1016/j.jacc.2017.08.004 28911515

[2] WHO. Chagas Disease (American Trypanosomiasis). World Health Organization: Fact sheet. 2017. Available at: http://www.who.int/mediacentre/factsheets/fs340/en/.

[3] Bestetti RB Santos CR Machado-Júnior OB Ariolli MT Carmo JL Costa NK. Clinical Profile of Patients With Chagas’ Disease Before and During Sustained Ventricular Tachycardia. Int J Cardiol (1990) 29:39–46. 10.1016/0167-5273(90)90271-6 2148167

[4] Bestetti RB Ariolli MT do Carmo JL Passos AD Santos CR Machado Júnior O. Clinical Characteristics of Acute Myocardial Infarction in Patients With Chagas Disease. Int J Cardiol (1992) 35:371–6. 10.1016/0167-5273(92)90236-V 1612801

[5] Teixeira ARL Nascimento RJ Sturm NR. Evolution and Pathology in Chagas Disease - a Review. Mem Inst Oswaldo Cruz (2006) 101:463–91. 10.1590/S0074-02762006000500001 17072450

[6] Bestetti RB. Role of Parasites in the Pathogenesis of Chagas’ Cardiomyopathy. Lancet (1996) 347:913–4. 10.1016/s0140-6736(96)91403-8 8622441

[7] de Oliveira RB Troncon LE Dantas RO Menghelli UG. Gastrointestinal Manifestations of Chagas’ Disease. Am J Gastroenterol (1998) 93:884–9. 10.1111/j.1572-0241.1998.270_r.x 9647012

[8] Cardillo F Pinho RT Antas PRZ Mengel J. Immunity and Immune Modulation in *Trypanosoma Cruzi* Infection. Pathog Dis (2015) 73:ftv082. 10.1093/femspd/ftv082 26438729 PMC4626602

[9] Jiménez P Jaimes J Poveda C Ramírez JD. A Systematic Review of the Trypanosoma Cruzi Genetic Heterogeneity, Host Immune Response and Genetic Factors as Plausible Drivers of Chronic Chagasic Cardiomyopathy. Parasitology (2018) 146:269–83. 10.1017/S0031182018001506 30210012

[10] Dutra WO Menezes CA Villani FN da Costa GC da Silveira AB Reis DD. Cellular and Genetic Mechanisms Involved in the Generation of Protective and Pathogenic Immune Responses in Human Chagas Disease. Mem Inst Oswaldo Cruz (2009) 104:208–18. 10.1590/S0074-02762009000900027 PMC3285444 19753476

[11] Benvenuti LA Roggerio A Freitas HF Mansur AJ Fiorelli A Higuchi ML. Chronic American Trypanosomiasis: Parasite Persistence in Endomyocardial Biopsies is Associated With High-Grade Myocarditis. Ann Trop Med Parasitol (2008) 102:481–7. 10.1179/136485908X311740 18782487

[12] Bonney KM Engman DM. Autoimmune Pathogenesis of Chagas Heart Disease: Looking Back, Looking Ahead. Am J Pathol (2015) 185:1537–47. 10.1016/j.ajpath.2014.12.023 PMC4450315 25857229

[13] Sathler-Avelar R Vitelli-Avelar DM Teixeira-Carvalho A Martins-Filho OA. Innate Immunity and Regulatory T-Cells in Human Chagas Disease: What Must be Understood? Mem Inst Oswaldo Cruz (2009) 104:246–51. 10.1590/S0074-02762009000900031 19753480

[14] Tarleton RL. CD8+ T Cells in Trypanosoma Cruzi Infection. Semin Immunopathol (2015) 37:233–8. 10.1007/s00281-015-0481-9 PMC4654118 25921214

[15] Brener Z Gazzinelli RT. Immunological Control of Trypanosoma Cruzi Infection and Pathogenesis of Chagas’ Disease. Int Arch Allergy Immunol (1997) 114:103–10. 10.1159/000237653 9338602

[16] d’Avila Reis D Lemos EM Silva GC Adad SJ McCurley T Correa-Oliveira R. Phenotypic Characterization of the Inflammatory Cells in Chagasic Megaoesophagus. Trans R Soc Trop Med Hyg (2001) 95:177–78. 10.1016/s0035-9203(01)90153-1 11355554

[17] da Silveira ABM Lemos EM Adad SJ Correa-Oliveira R Furness JB Reis DA. Megacolon in Chagas Disease: A Study of Inflammatory Cells, Enteric Nerves, and Glial Cells. Hum Pathol (2007) 38:1256–64. 10.1016/j.humpath.2007.01.020 17490721

[18] Bauer S Groh V Wu J Steinle A Phillips JH Lanier LL. Activation of NK Cells and T Cells by NKG2D, a Receptor for Stress-Inducible MICA. Science (1990) 285:727–9. 10.1126/science.285.5428.727 10426993

[19] Lanier LL. NK Cell Recognition. Annu Rev Immunol (2005) 23:225–74. 10.1146/annurev.immunol.23.021704.115526 15771571

[20] Zwirner NW Dole K Stastny P. Differential Surface Expression of MICA by Endothelial Cells, Fibroblasts, Keratinocytes, and Monocytes. Hum Immunol (1999) 60:323–30. 10.1016/s0198-8859(98)00128-1 10363723

[21] Groh V Rhinehart R Randolph-Habecker J Topp MS Riddell SR Spies T. Costimulation of CD8alphabeta T Cells by NKG2D *via* Engagement by MIC Induced on Virus-Infected Cells. Nat Immunol (2001) 2:255–60. 10.1038/85321 11224526

[22] Pardoll DM. Stress, NK Receptors, and Immune Surveillance. Science (2001) 294:534–6. 10.1126/science.1066284 11567108

[23] Ljunggren HG Kärre K. In Search of the “Missing Self”: MHC Molecules and NK Cell Recognition. Immunol Today (1990) 11:237–44. 10.1016/0167-5699(90)90097-s 2201309

[24] Middleton D Gonzelez F. The Extensive Polymorphism of KIR Genes. Immunology (2010) 129:8–19. 10.1111/j.1365-2567.2009.03208.x 20028428 PMC2807482

[25] del Puerto F Nishizawa JE Kikuchi M Roca Y Avilas C Gianella A. Protective Human Leucocyte Antigen Haplotype, HLA-DRB1*01-B*14, Against Chronic Chagas Disease in Bolivia. PloS Negl Trop Dis (2012) 6:e1587. 10.1371/journal.pntd.0001587 22448298 PMC3308929

[26] Ayo CM de Oliveira AP Camargo AV Brandão de Mattos CC Bestetti RB de Mattos LC. Association of the Functional MICA-129 Polymorphism With the Severity of Chronic Chagas Heart Disease. Clin Infect Dis (2015) 61:1310–3. 10.1093/cid/civ540 PMC4583584 26129751

[27] Ayo CM Reis PG Dalalio MM Visentainer JE Oliveira C de F de Araújo SM. Killer Cell Immunoglobulin-Like Receptors and Their HLA Ligands are Related With the Immunopathology of Chagas Disease. PloS Negl Trop Dis (2015) 9:e0003753. 10.1371/journal.pntd.0003753 25978047 PMC4433128

[28] Little J Higgins JP Ioannidis JP Moher D Gagnon F von Elm E. Strengthening the Reporting of Genetic Association Studies (STREGA): An Extension of the STROBE Statement. PLoS Med (2009) 6:e1000022. 10.1371/journal.pmed.1000022 PMC2634792 19192942

[29] Parra FC Amado RC Lambertucci JR Rocha J Antunes CM Pena SDJ. Color and Genomic Ancestry in Brazilians. Proc Nat Aca Sci (2003) 100:177–82. 10.1073/pnas.0126614100 PMC140919 12509516

[30] Bestetti RB Otaviano AP Cardinalli-Neto A da Rocha BF Theodoropoulos TA Cordeiro JA. Effects of B-Blockers on Outcome of Patients With Chagas’ Cardiomyopathy With Chronic Heart Failure. Int J Cardiol (2011) 151:205–8. 10.1016/j.ijcard.2010.05.033 20591516

[31] Dias JC Ramos AN Jr Gontijo ED Luquetti A Shikanai-Yasuda MA Coura JR. 2nd Brazilian Consensus on Chagas Disease, 2015. Rev Soc Bras Med Trop (2016) 49:3–60. 10.1590/0037-8682-0505-2016 27982292

[32] Carr WH Pando MJ Parham P. KIR3DL1 Polymorphisms That Affect NK Cell Inhibition by HLA-Bw4 Ligand. J Immunol (2005) 175:5222–9. 10.4049/jimmunol.175.8.5222 16210627

[33] Thananchai H Gillespie G Martin MP Bashirova A Yawata N Yawata M. Allele-Specific and Peptide-Dependent Interactions Between KIR3DL1 and HLA-A and HLA-B. J Immunol (2007) 178:33–7. 10.4049/jimmunol.178.1.33 17182537

[34] Kulkarni S Martin MP Carrington M. The Yin and Yang of HLA and KIR in Human Disease. Semin Immunol (2008) 20:343–52. 10.1016/j.smim.2008.06.003 PMC3501819 18635379

[35] Steinle A LIP Morris DL Groh V Lanier LL Strong RK Spies T. Interactions of Human NKG2D With its Ligands MICA, MICB, and Homologs of the Mouse RAE-1 Protein Family. Immunogenetics (2001) 53:279–87. 10.1007/s002510100325 11491531

[36] Karacki PS Gao X Thio CL Thomas DL Goedert JJ Vlahov D. MICA and Recovery From Hepatitis C Virus and Hepatitis B Virus Infections. Genes Immun (2004) 5:261–6. 10.1038/sj.gene.6364065 15029237

[37] Guo SW Thompson EA. Performing the Exact Test of Hardy Weinberg Proportion for Multiple Alleles. Biometrics (1992) 48:361–72. 10.2307/2532296 1637966

[38] Imanishi T Akaza T Kimura A Tokunaga K Gojobori T. “Allele and Haplotype Frequencies for HLA and Complement Loci in Various Ethnic Groups”. In: Tsuji K Aizawa M Sasazuki T, editors. HLA 1991: Proceedings of the Eleventh International Histocompatibility Workshop and Conference. Oxford, UK: Oxford University Press (1992). p. 1065–220.

[39] Shifrin N Raulet DH Ardolino M. NK Cell Self Tolerance, Responsiveness and Missing Self Recognition. Semin Immunol (2014) 26:138–44. 10.1016/j.smim.2014.02.007 PMC3984600 24629893

[40] Lanier LL. NKG2D Receptor and Its Ligands in Host Defense. Cancer Immunol Res (2015) 3:575–82. 10.1158/2326-6066.CIR-15-0098 PMC4457299 26041808

[41] Cunha-Neto E Chevillard C. Chagas Disease Cardiomyopathy: Immunopathology and Genetics. Mediators Inflamm (2014) 2014:683230. 10.1155/2014/683230 25210230 PMC4152981

[42] Gazzinelli RT Oswald IP Hieny S James SL Sher A. The Microbicidal Activity of Intreferon-G Treated Macrophages Against Tripanosoma Cruzi Involves an Larginine-Dependent, Nitrogen Oxide-Mediared Mechanism Inhibitable by Interleukin-10 and Transforming Growth Factor-B. Euro J Immunol (1992) 22:2501–6. 10.1002/eji.1830221006 1396957

[43] Teixeira ARL Hecht MM Guimaro MC Sousa AO Nitz N. Pathogenesis of Chagas’ Disease: Parasite Persistence and Autoimmunity. Clin Microbiol Rev (2011) 24:592–630. 10.1128/CMR.00063-10 21734249 PMC3131057

[44] Corbett CE Ribeiro U Prianti MG Habr-Gama A Okumura M Gama-Rodrigues J. Cell-Mediated Immune Response in Megacolon From Patients With Chronic Chagas’ Disease. Dis Colon Rectum (2001) 44:993–8. 10.1007/bf02235488 11496080

[45] Suemizu H Radosavljevic M Kimura M Sadahiro S Yoshimura S Bahram S. A Basolateral Sorting Motif in the MICA Cytoplasmic Tail. Proc Natl Acad Sci USA (2002) 99:2971–6. 10.1073/pnas.052701099 PMC122457 11854468

[46] Groh V Bruhl A El-Gabalawy H Nelson JL Spies T. Stimulation of T Cell Autoreactivity by Anomalous Expression of NKG2D and its MIC Ligands in Rheumatoid Arthritis. Proc Natl Acad Sci USA (2003) 100:9452–7. 10.1073/pnas.1632807100 PMC170939 12878725

[47] Hüe S Mention JJ Monteiro RC Zhang S Cellier C Schmitz J. Direct Role for NKG2D/MICA Interaction in Villous Atrophy During Celiac Disease. Immunity (2004) 21:367–277. 10.1016/j.immuni.2004.06.018 15357948

[48] Bahram S Inoko H Shiina T Radosavljevic M. MIC and Other NKG2D Ligands: From None to Too Many. Curr Opin Immunol (2005) 17:505–9. 10.1016/j.coi.2005.07.016 16087327

[49] Groh V Steinle A Bauer S Spies T. . Recognition of Stress-Induced MHC Molecules by Intestinal Epithelial Gammadelta T Cells. Science (1998) 279:1737–40. 10.1126/science.279.5357.1737 9497295

[50] Wong P Pamer EG. CD8 T-Cell Responses to Infectious Pathogens. Annu Rev Immunol (2003) 21:29–70. 10.1146/annurev.immunol.21.120601.141114 12414723

[51] Stephens HAF. MICA and MICB Genes: Can the Enigma of Their Polymorphism be Resolved? Trends Immunol (2001) 22:378–85. 10.1016/s1471-4906(01)01960-3 11429322

[52] Williams AP Bateman AR Khakoo SI. Hanging in Balance. KIR and Their Role in Disease. Mol Interv (2005) 5:226–40. 10.1124/mi.5.4.6 16123537

[53] Khakoo SI Carrington M. KIR and Disease: A Model System or System of Models? Immunol Rev (2006) 214:186–201. 10.1111/j.1600-065X.2006.00459.x 17100885

[54] Moesta AK Parham P. Diverse Functionality Among Human NK Cell Receptors for the C1 Epitope of HLA-C: KIR2DS2, KIR2DL2, and KIR2DL3. Front Immunol (2012) 22:336. 10.3389/fimmu.2012.00336 PMC3504360 23189078

[55] Hollenbach JA Ladner MB Saeteurn K Taylor KD Mei L Haritunians T. Susceptibility to Crohn’s Disease is Mediated by *KIR*2DL2/*KIR*2DL3 Heterozygosity and the HLA-C Ligand. Immunogenetics (2009) 61:663–71. 10.1007/s00251-009-0396-5 PMC2813946 19789864

[56] Jones DC Edgar RS Ahmad T Cummings JR Jewell DP Trowsdale J. Killer Ig-Like Receptor (KIR) Genotype and HLA Ligand Combinations in Ulcerative Colitis Susceptibility. Genes Immun (2006) 7:576–82. 10.1038/sj.gene.6364333 16929347

[57] Vivier E Raulet DH Moretta A Caligiuri MA Zitvogel L Lanier LL. Innate or Adaptive Immunity? The Example of Natural Killer Cells. Science (2011) 331:44–9. 10.1126/science.1198687 PMC3089969 21212348

[58] Vitelli-Avelar DM Sathler-Avelar R Massara RL Borges JD Lage PS Lana M. Are Increased Frequency of Macrophage-Like and Natural Killer (NK) Cells, Together With High Levels of NKT and CD4+CD25high T Cells Balancing Activated CD8+ T Cells, the Key to Control Chagas’ Disease Morbidity? Clin Exp Immunol (2006) 145:81–92. 10.1111/j.1365-2249.2006.03123.x 16792677 PMC1942003

[59] Martins PRJ Martins RA Barbosa V de F Pereira G de A Moraes-Souza H Silva SS. The Importance of Hemovigilance in the Transmission of Infectious Diseases. Rev Bras Hematol Hemoter. Associação Bras Hematol e Hemoterapia (2013) 35:180–4. 10.5581/1516-8484.20130040 PMC3728130 23904807

[60] Golding J Northstone K Miller LL Davey Smith G Pembrey M. Differences Between Blood Donors and a Population Sample: Implications for Case-Control Studies. Int J Epidemiol (2013) 42:1145–56. 10.1093/ije/dyt095 PMC3781001 23825379

